# THE HEALTHY AGING INITIATIVE: LONGITUDINAL RESEARCH EMPOWERING OLDER ADULTS TO LIVE THEIR BEST LIVES

**DOI:** 10.1093/geroni/igad104.3644

**Published:** 2023-12-21

**Authors:** Heather Andrews, Talia Gilfix, Benjamin Stein, Davide Cappon, Maggie Syme, Alvaro Pascual-Leone

**Affiliations:** Hebrew SeniorLife, Boston, Massachusetts, United States; Hebrew SeniorLife, Boston, Massachusetts, United States; Hebrew Rehabilitation Center for the Aged, Boston, Massachusetts, United States; Hebrew Rehabilitation Center for the Aged, Boston, Massachusetts, United States; Hebrew Rehabilitation Center for the Aged, Boston, Massachusetts, United States; Hebrew Rehabilitation Center for the Aged, Boston, Massachusetts, United States

## Abstract

The World Health Organization (WHO) has designated this the decade of Healthy Ageing--focused on developing and maintaining functional ability that enables well-being in older age. We developed the Healthy Aging Initiative (HAI), a prospective cohort study of adults 55+ measuring functional ability, going beyond a disease-focused model of population ageing to holistic intrinsic capacity (physical, cognitive, emotional, behavioral, cultural health) and environmental context. A multidisciplinary team of geroscientists developed a protocol of subjective and objective assessments evaluating several known modifiable risk factors for disability and dementia, mobility, strength, functional status, and mental wellness. By tracking these domains annually, we influence participant health trajectories with timely, actionable feedback and gain insight into causal factors that impact functional ability, creating a blueprint for translational clinical research in healthy ageing. A feasibility pilot (N=37; 80.8 avg. age, 67-93 age range; 92% White) was conducted within two affiliated older adult living communities. The revision process included evaluating specificity, sensitivity, and utility of all assessments (results to be presented), and accounting for participant suggestions (results to be presented). The majority of pilot participants perceived benefit from the assessment (81%) and would participate again (86%), despite 43% reporting the length as challenging. Results were used to revise the protocol content and process, including reducing assessment time from 2+ hours to 40 minutes, and completing surveys over the phone. We have begun the full roll out (HAI 2.0) into all affiliated older adult living communities (2023), and will have second annual assessments in the upcoming months.

